# Psycho-oncological burden in patients with brain metastases undergoing neurological surgery

**DOI:** 10.3389/fonc.2024.1463467

**Published:** 2024-11-28

**Authors:** Tommaso Araceli, Anna Fischl, Amer Haj, Christian Doenitz, Eva-Maria Stoerr, Andrea Hillberg, Martin Vogelhuber, Katharina Rosengarth, Markus J. Riemenschneider, Peter Hau, Raquel Blazquez, Tobias Pukrop, Elisabeth Bumes, Nils Ole Schmidt, Martin Proescholdt

**Affiliations:** ^1^ Department of Neurosurgery, Regensburg University Medical Center, Regensburg, Germany; ^2^ Wilhelm Sander-NeuroOncology Unit, Regensburg University Medical Center, Regensburg, Germany; ^3^ Department of Neurology, Regensburg University Medical Center, Regensburg, Germany; ^4^ Department of Internal Medicine III, Regensburg University Medical Center, Regensburg, Germany; ^5^ Department of Neuropathology, Regensburg University Medical Center, Regensburg, Germany; ^6^ Bavarian Cancer Research Center (BZKF), Regensburg, Germany

**Keywords:** psycho-oncology, brain tumor, neurosurgery, psychological distress, psycho-oncological need

## Abstract

**Purpose:**

The development of brain metastases (BM) can significantly increase the psycho-oncological burden in cancer patients, requiring timely intervention. In addition, this aspect may negatively affect the course of the disease and treatment outcome. However, screening for psycho-oncological burden is often overlooked in clinical routine. Therefore, we analyzed the extent of psycho-oncological distress in a patient population with BM receiving neurosurgical resection and identified clinical characteristics associated with a high need for psycho-oncological intervention.

**Methods:**

We prospectively screened 353 patients (169 female, 184 male, mean age 61.9 years) scheduled for microsurgical resection of one or more BM. Psycho-oncological screening was performed on the day of admission using the Hornheider screening instrument (HSI) and the distress thermometer (DT). Screening results were correlated with demographic and clinical data.

**Results:**

Most patients (73.1%) completed the screening questionnaire. Patients who failed to complete the questionnaire presented more frequently with metachronous BM (74.7% *vs*. 25.3%, p=0.009), were significantly older (p=0.0018), and had a significantly lower KPS score (p=0.0002). Based on the threshold values of the questionnaires, 59.3% of the patients showed a significant psycho-oncological burden requiring immediate intervention. Univariate analysis demonstrated that synchronous BM (p=0.034), tumors in eloquent areas (p=0.001), lower KPS (p=0.031), female gender (p=0.009), and presurgical aphasia (p=0.042) were significantly associated with high psycho-oncological burden. Multivariate analysis showed synchronous BM (p=0.045), female gender (p=0.005), and lower KPS (p=0.028) as independent factors associated with high psycho-oncological burden.

**Conclusion:**

The majority of patients with BM have a high psycho-oncological burden. Female gender, synchronous BM, and lower KPS are independently associated with a need for psycho-oncological intervention.

## 1 Introduction

The development of brain metastases (BM) can significantly worsen the prognosis of patients with cancer (1) and is an increasingly common complication of the primary disease (2). Patients with BM are severely burdened by metastasis-related symptoms and the exceptionally poor prognosis (3). As a life-threatening disease, cancer increases the risk of developing mental health problems, including depression, anxiety, and distress (4). These factors have been shown to be significant determinants of quality of life (QoL) (5). Depression and anxiety in particular negatively influence treatment outcome and survival (6). The National Comprehensive Cancer Network (NCCN) defines distress in cancer as “a multifactorial unpleasant experience of a psychological, social, spiritual, and/or physical nature that may interfere with one’s ability to cope effectively with cancer, its physical symptoms, and its treatment” (7). Therefore, patients with a high level of distress need supportive care and psycho-oncological intervention (8). It is highly important that each individual patient in need of psycho-oncological support is identified correctly and in a timely manner (9). Two well-established tools, the Hornheider Screening Instrument (HSI) and the Distress Thermometer (DT), can be used to assess psycho-oncological distress (10–18). The HSI is an appropriate tool with high reliability and validity using the answer categories “yes” and “no” to assess the physical and mental status of patients during the initial contact between physicians and patients (19). DT is recognized as a brief, feasible, and highly sensitive screening tool when evaluated against established criteria (17, 20, 21). However, with the exception of specialized neuro-oncology centers, screening for psycho-oncological distress is not regularly established in the clinical routine of neurosurgical units, and the need for psycho-oncological support may often be underestimated (22). We hypothesized that patients with BM and high psycho-oncological needs may be identified by specific characteristics such as older age, low KPS, or focal neurological impairment. The aim of this study was therefore to analyze the subgroup of patients with BM scheduled for neurological surgery who showed increased psycho-oncological burden, in order to identify clinical parameters that predict this specific unmet medical need. Although similar studies have been performed in patients receiving radiotherapy (23–25) or systemic treatment (26), no such analysis has yet been performed in patients with BM undergoing microsurgical resection.

## 2 Material and methods

### 2.1 Study design and ethical approval

This single-center cross-sectional study prospectively enrolled patients scheduled for microsurgical resection of one or more BM at the Regensburg Brain Tumor Center between January 2015 and January 2023. After being informed about the objectives of the study and confirming the voluntary participation, patients were questioned once at first admission using the HSI or the DT and divided into two groups with and without the need for psycho-oncological care.

In accordance with German ethical and regulatory standards and the Declaration of Helsinki (7^th^ revision, 2013), the study was approved by the Regensburg University Institutional Ethics Review Board (vote no. 20-1799-101). The data protection concept at the Brain Tumor Center Regensburg, established according to the European General Data Protection Regulation and relevant national legislation, was strictly followed.

### 2.2 Questionnaires

The HSI is a questionnaire designed to assess psycho-oncological needs of cancer patients. It contains 7 items that examine global health conditions, global mental conditions, burden, person of trust, burdened family member, temporary internal disturbance, and information about the disease and treatment. The individual items are aggregated into a summary score ranging from 0 to 14. The cut-off is set at 5 score points, with scores ≥ 5 points indicating the need for psycho-oncological support (11). The DT is a screening instrument developed by the NCCN Distress Management Panel to provide an initial screening of psycho-oncological distress in cancer patients. Its scale is 0 to 10, and a score greater than 4 indicates psycho-oncological need (27).

The psycho-oncological screening was performed on the day of admission. Examples of the questionnaires are attached in the **Supplementary Files** (**Supplementary Material 1**). The questionnaire given to the patients was selected according to the hospital’s internal standards. The change from HSI to DT was based on a consensus decision made by the leading board of the local Comprehensive Cancer Centers network in Würzburg, Erlangen, Regensburg, and Augsburg (CCC – WERA), aligning with the current guidelines (28). Accordingly, we have implemented this decision into our clinical practice. A value of ≥ 5 in the HSI or > 4 in the DT indicated high psycho-oncological distress.

### 2.3 Study population

During the patient recruitment phase, data on the entire cohort were filtered out. Inclusion criteria were admission to the neurosurgical department because of suspected brain metastasis or known primary systemic oncologic disease and presence of an intracerebral tumor mass on MRI, an appropriate recruiting time frame before neurosurgical resection, age older than 18 years, and histological confirmation of the diagnosis BM after the resection. Patients without psycho-oncological screening at admission or with ambiguous or unclear answers were excluded.

The following variables were collected from the electronic patient files of the SAP^®^ software (SAP^®^ Deutschland SE & Co.KG, Walldorf, Germany) and the radiological, oncological, medical, and tumor board reports: age, gender, preoperative Karnofsky Performance Status (KPS), tumor-related deficits, histology of the primary tumor, BM timing, side and location of the BM, BM status (solitary = one single BM without systemic metastases, singular = one singular BM and at least one systemic metastasis, and multiple = more than one BM), and extent of resection. Eloquent areas were defined using a widely used summary description in the literature that describes eloquent cerebral structures as brain areas with readily identifiable neurological function, where injury results in disability (29).

### 2.4 Statistics

For continuous data, descriptive statistics were applied (Stata/IC version 16.1, College Station, USA) using mean, median, minimum, maximum, and standard deviation. Categorical data are presented as absolute and relative frequencies. Continuous variables were compared using the Student’s t-test for normally distributed data and the Mann-Whitney U test for non-normally distributed data. A multivariate analysis was performed using a multiple linear regression model, and the independence of categorical variables was tested with Pearson’s chi-squared. A p-value < 0.05 was defined as statistically significant.

## 3 Results

### 3.1 Population characteristics

**Figure 1:**
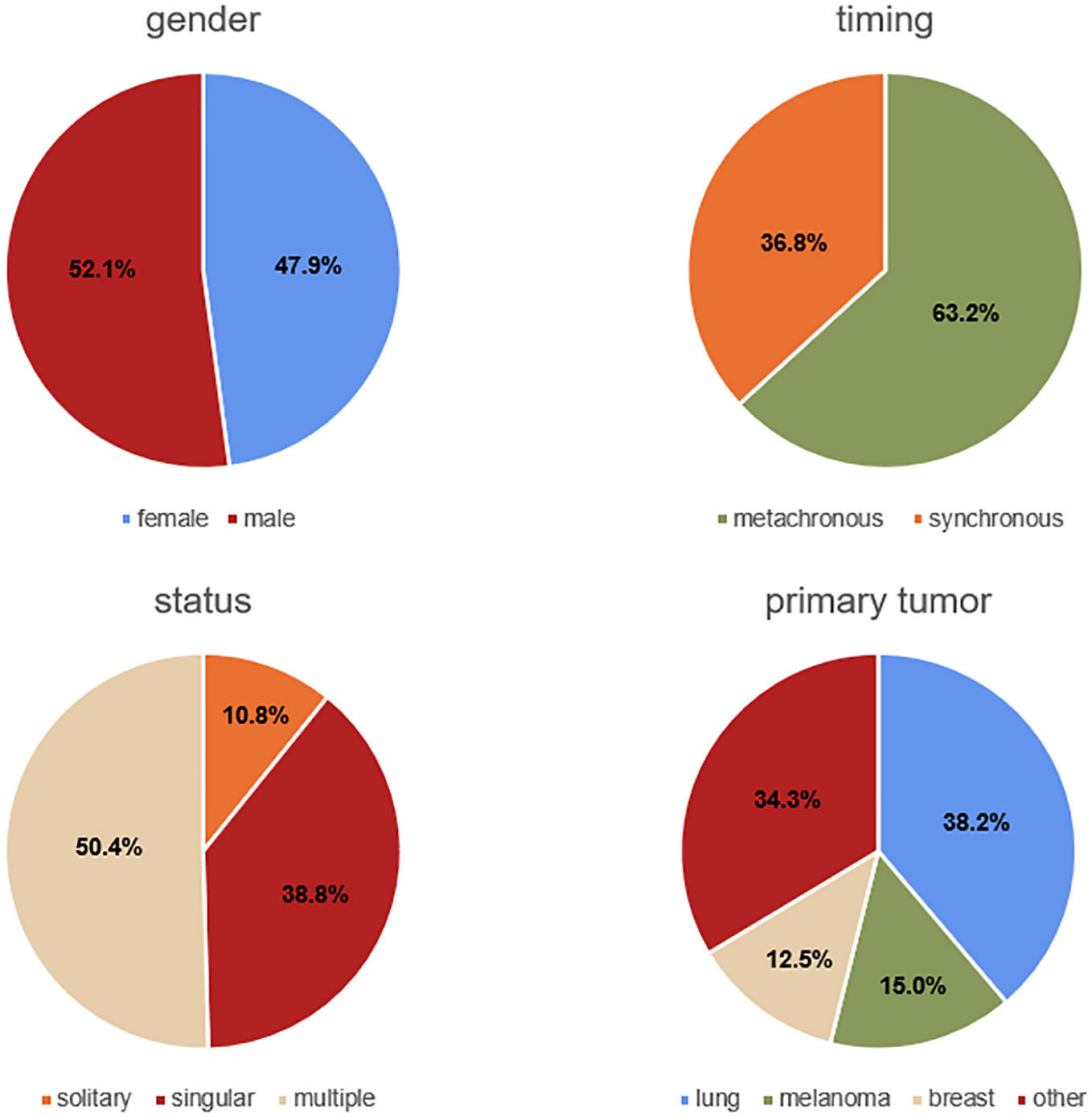
Pie charts showing part of baseline data.

Our study included 353 patients (169 female and 184 male between the ages of 26.3 and 85.1 years, mean age 61.9 ± 12.2 years). 186 patients who did not meet the inclusion criteria were excluded. In the recruited population, the mean preoperative KPS was 79.5 ± 15.7 (range: 30-100). 50.4% (n=178) of the patients presented with multiple metastases, 38.8% (n=137) with singular, and 10.8% (n=38) with solitary metastasis. The majority of the patients (63.2%, n=223) were treated for metachronous metastases, and the remaining patients for synchronous metastases (36.8%, n=130). The most frequent primary tumor was lung cancer (38.2%, n=135), followed by melanoma (15.0%, n=53), and breast cancer (12.5%, n=44). Complete resection was achieved in 78.5% (n=277) of the patients, while resection was incomplete in 21.5% (n=76). 47 (13.3%) patients were affected by aphasia, 77 (21.8%) showed hemiparesis, and 49 (13.9%) had visual impairments. Regarding the anatomical site of the lesion, 113 (32.0%) were frontal, 78 (22.1%) cerebellar, 57 (16.1%) parietal, 51 (14.5%) occipital, 39 (11.0%) temporal, 9 (2.6%) frontoparietal, and 6 (1.7%) frontotemporal. In total, 155 (43.9%) lesions were located on the right side, 151 (42.8%) on the left side, and 47 (13.3%) were bilateral. 114 (32.3%) were situated in an eloquent area. The baseline data are summarized in **Table 1** and partially illustrated in **Figure 1**.

**Table 1:** Baseline data.

Parameter	Value
Total population	353
Gender (m/f)	184/169 (52.1/47.9)
Age	61.9 (range: 26.3–85.1)
Preoperative KPI	80 (range: 30–100)
Metastasis status	Solitary: 38 (10.8) Singular: 137 (38.8) Multiple: 178 (50.4)
Metastasis timing	Synchronous: 130 (36.8) Metachronous: 223 (63.2)
Primary	Lung: 135 (38.2) Melanoma: 53 (15.0) Breast: 44 (12.5) Colorectal: 24 (6.8) CUP: 23 (6.5) Kidney: 13 (3.7) Stomach: 8 (2.3) Prostate: 8 (2.3) Urothelium: 7 (1.9) Endometrium: 6 (1.7) Cervix: 5 (1.4) Testis: 2 (0.6) Other: 25 (7.1)
Deficits	
- Hemiparesis - Visual impairment - Aphasia	77 (21.8) 49 (13.9) 47 (13.3)
Localization	
- Frontal - Cerebellar - Parietal - Occipital - Temporal - Frontoparietal - Frontotemporal	113 (32.0) 78 (22.1) 57 (16.1) 51 (14.5) 39 (11.0) 9 (2.6) 6 (1.7)
Side	
- Right - Left - Bilateral	155 (43.9) 151 (42.8) 47 (13.3)
Eloquent area	114 (32.3)
Complete resection (y/n)	277 (78.5)/76 (21.5)

Values are given as number of patients (%) or median (range).

### 3.2 Completion of questionnaires

**Figure 2:**
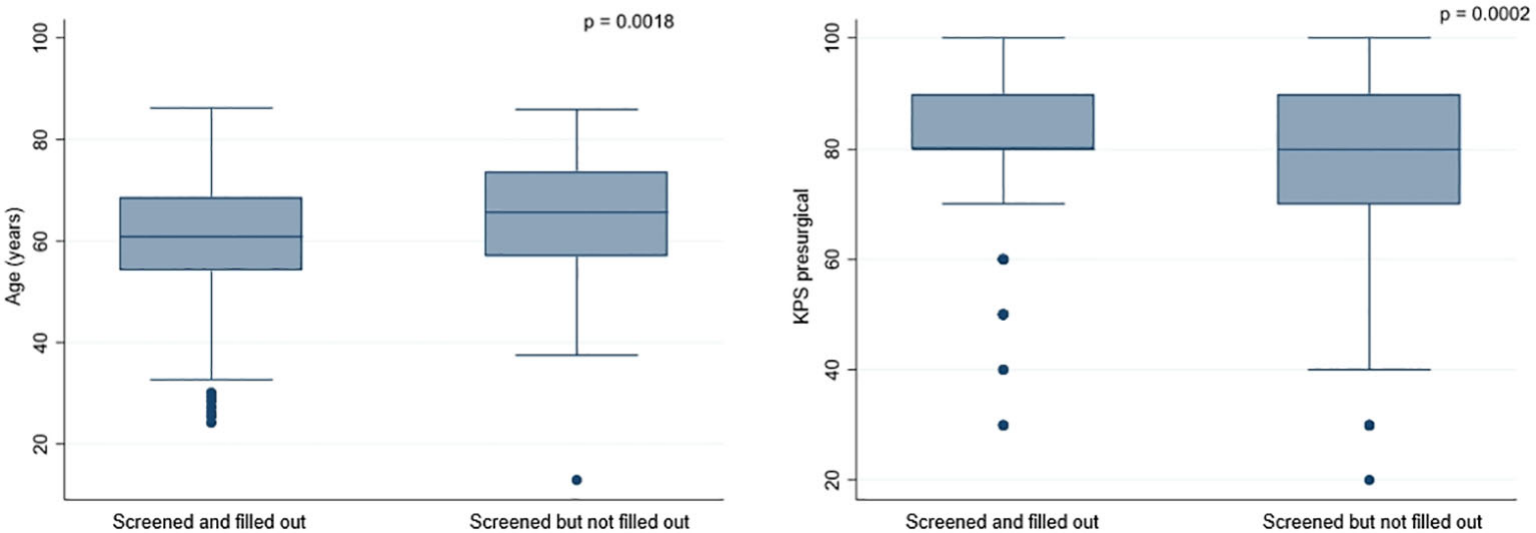
Graphs illustrating the significant influence of age and presurgical KPS on questionnaire completion.

Most patients (258, 73.1%) completed the screening questionnaire. This subgroup showed a mean age of 60.6 ± 12 years and a preoperative mean KPS of 81.4 ± 13.8. 152 (58.9%) patients had metachronous, and 106 (41.1%) patients had synchronous metastasis timing. Psycho-oncological screening using the HSI was performed in 241 (93.4%) patients and with the DT in 17 (6.6%) patients. 95 (26.9%) patients failed to complete the questionnaire. The characteristics of this subpopulation were as follows: mean age 65.4 ± 12.2 years, mean preoperative KPS 74.4 ± 19, 71 (74.7%) patients with metachronous presentation and 24 (25.3%) patients with synchronous presentation. Univariate analysis showed that the patients who failed to complete the questionnaire were significantly older (60.6 *vs*. 65.4, p=0.0018), presented significantly more frequently with metachronous BM (74.7 *vs*. 25.3%, p=0.009), and showed a significantly lower presurgical KPS (74.4 *vs*. 81.4, p=0.0002) than patients who filled out the questionnaire. These results are illustrated in **Table 2** and **Figure 2**.

**Table 2:** Influence of BM timing on questionnaire completion.

Metastasis timing	Screening failure		p-value
	no = 258 (73.1)	yes = 95 (26.9)	
Synchronous	106 (41.1)	24 (25.3)	**0.009**
Metachronous	152 (58.9)	71 (74.7)	

Values are given as number of patients (%). The p-value is highlighted in bold.

### 3.3 Psycho-oncological need

**Figure 3:**
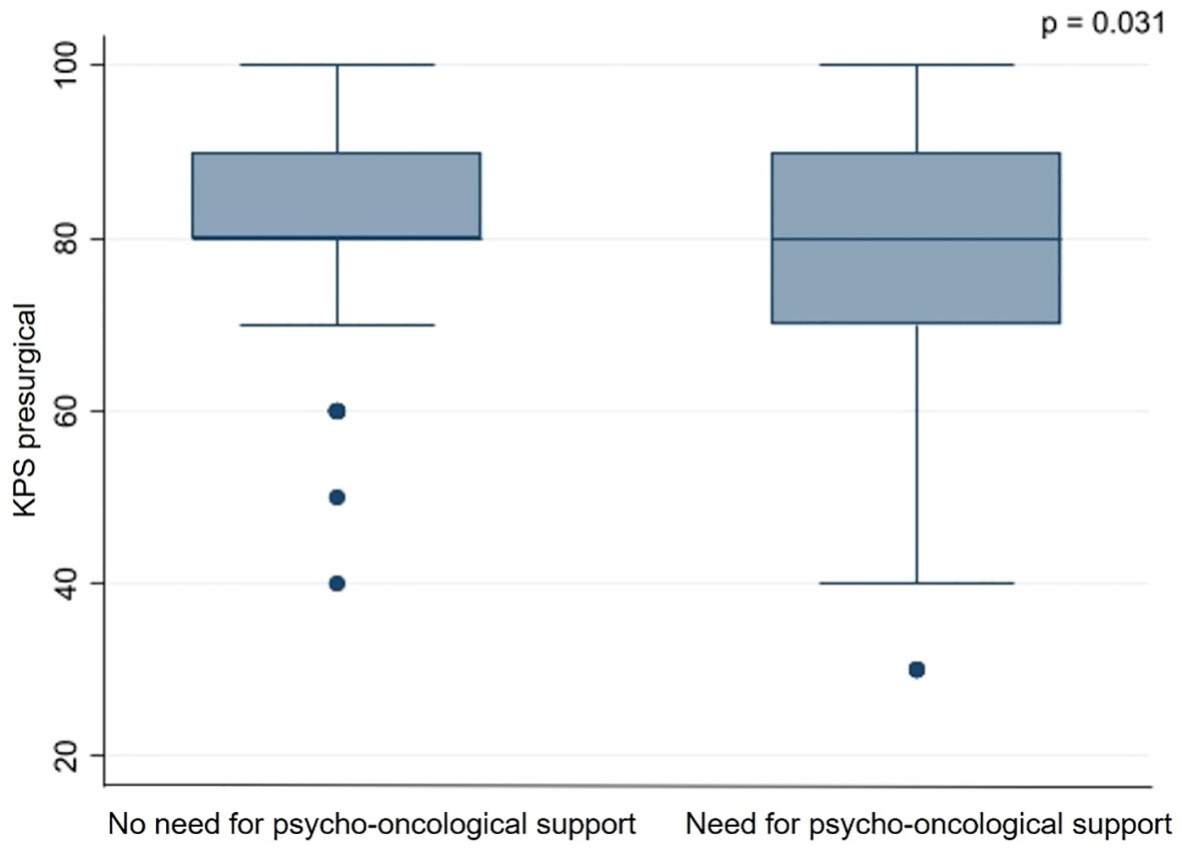
Influence of presurgical KPS on psycho-oncological burden.

**Figure 4:**
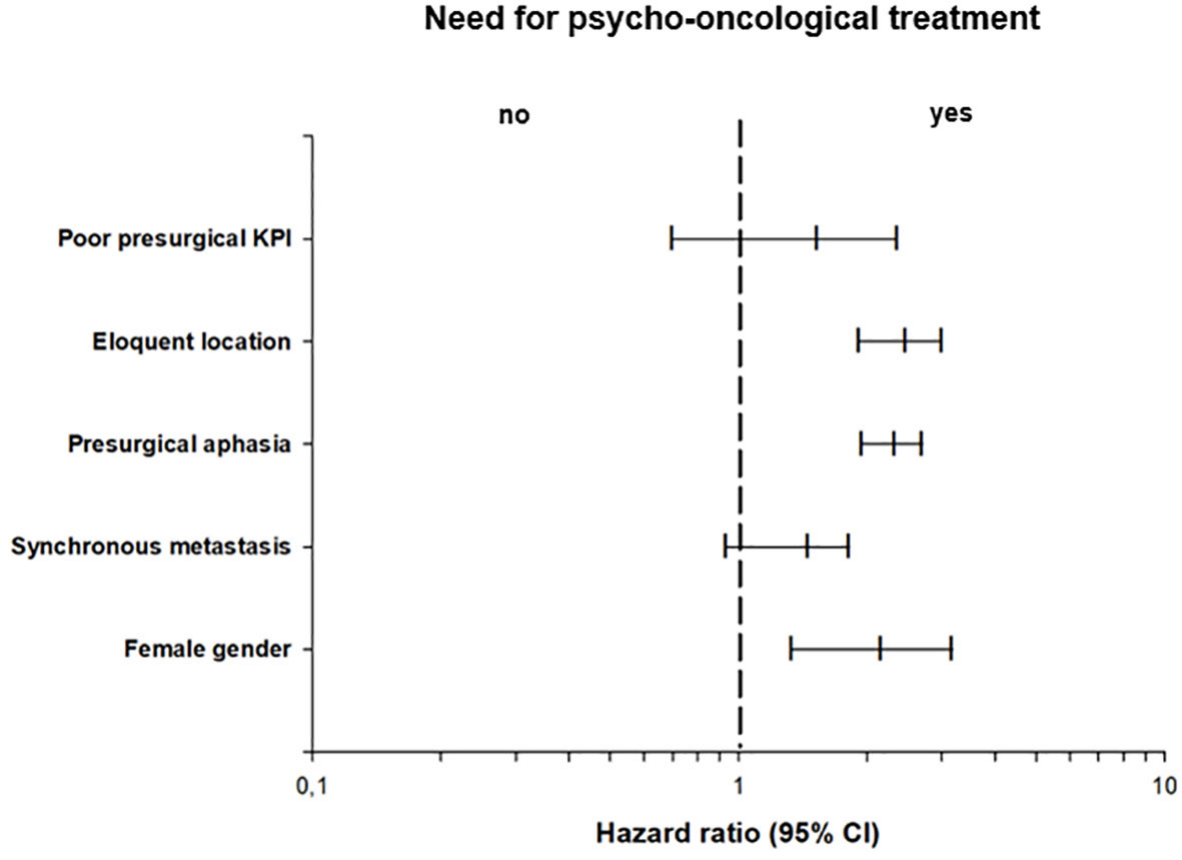
Multivariate binary logistic regression analysis of psycho-oncological need, showing the odds ratios of the impact of clinical characteristics in relation to psycho-oncological burden.

Based on the thresholds of the questionnaires, 153 (59.3%) patients showed a significant psycho-oncological burden requiring immediate intervention, while 105 (40.7%) patients did not 85 (55.6%) female and 68 (44.4%) male patients required psycho-oncological care, while 64 (60.9%) male and 41 (39.1%) female patients did not need psycho-oncological support. According to the univariate analysis psycho-oncological need was significantly higher in female gender (p=0.009). 96 (62.7%) patients with high psycho-oncological distress had BM in a non-eloquent area, while 57 (37.3%) patients had BM in an eloquent area. In contrast, 86 (81.9%) patients with a distress value below the threshold had a non-eloquent BM, compared to the remaining 19 (18.1%) patients with BM in an eloquent area. Furthermore, 129 (84.3%) patients without aphasia and 24 (15.7%) patients with aphasia showed psycho-oncological distress values above the threshold. In comparison, 98 (93.3%) patients without aphasia and 7 (6.7%) patients with aphasia did not reach distress values above the threshold. When considering the timing of BM, 93 (60.8%) patients with metachronous BM and 60 (39.2%) with synchronous BM had above-normal distress values, in contrast to 78 (74.3%) patients with metachronous BM and 27 (25.7%) patients with synchronous BM who did not. Patients with high psycho-oncological distress had a lower KPS (80.1 ± 14.5) than the patients with a distress value below the threshold (83.3 ± 12.5). Univariate analysis thus demonstrated that tumors in an eloquent area (p=0.001), occurrence of aphasia (p=0.042), synchronous BM (p=0.034), and lower presurgical KPS (p=0.031) were significantly associated with high psycho-oncological burden (**Table 3**; **Figure 3**). The other variables did not significantly differ between the patients with or without high psycho-oncological distress. The multivariate analysis using a multiple linear regression model, showed that female gender (p=0.005), presurgical KPS (p=0.028), and synchronous BM (p=0.045) are independent factors associated with a high need for psycho-oncological support (**Table 4**; **Figure 4**).

**Table 3:** Influence of various clinical parameters on psycho-oncological need.

	Psycho-oncological need		p-value
Parameter	Beyond threshold = 153 (59.3)	Below threshold = 105 (40.7)	
Tumor location			
Non – eloquent	96 (62.7)	86 (81.9)	**0.001**
Eloquent	57 (37.3)	19 (18.1)	
Aphasia			
No Aphasia	129 (84.3)	98 (93.3)	**0.042**
Aphasia	24 (15.7)	7 (6.7)	
Metastasis timing			
Metachronous	93 (60.8)	78 (74.3)	**0.034**
Synchronous	60 (39.2)	27 (25.7)	
Gender			
Male	68 (44.4)	64 (60.9)	**0.009**
Female	85 (55.6)	41 (39.1)	

Values are given as number of patients (%). P-values are highlighted in bold.

**Table 4:** Multivariate analysis showing factors independently associated with a need for psycho-oncological intervention.

Parameter	Hazard Ratio	95% CI	p-value
Presurgical aphasia	2.325	2.703 1.948	0.317
Eloquent location	2.464	3.004 1.924	0.160
Female gender	2.668	3.173 2.163	**0.005**
Presurgical KPS	1.528	2.356 0.669	**0.028**
Synchronous metastasis	1.459	1.982 0.936	**0.045**

P-values less than or equal to 0.05 are highlighted in bold.

## 4 Discussion

This study evaluated for the first time the psycho-oncological burden in patients with BM receiving neurosurgical resection. Based on the results of the HSI and DT screening tools, this study has shown which subgroups of patients are most at risk and therefore may require more rapid and targeted psycho-oncological intervention.

### 4.1 Psycho-oncological burden in study populations

A survey of 4664 cancer patients treated at 55 American Cancer Centers demonstrated a significant psychological burden in 46% of all patients included (30). In contrast, in another study by Zabora et al. (2001), the overall prevalence rate of distress in patients with all types of cancer was only 35.1% (31). Patients with pancreatic or lung cancer as the primary tumor were associated with higher psycho-oncological burden (30, 31). To the best of our knowledge, there is not head-to-head study on whether cancer patients with BM have a higher psycho-oncological burden than those without BM. However, psychological distress, depression, and anxiety may be particularly enhanced in patients with primary brain tumors as compared to patients with non-CNS tumors (32, 33). Nevertheless, the literature shows considerable heterogeneity regarding the rate of psycho-oncological burden in patients with primary brain tumors, as shown in a recent meta-analysis, in which the prevalence of distress ranged from 12.3% to 73.6% (34). This extensive variability may be associated with the type of tumors and their different grades of malignancy. For example, a study on low-grade glioma showed a significant psycho-oncological burden in only 20.8% of the patients (35), whereas a similar study performed in patients with high-grade glioma found a rate of 61.5% (36). Those results are comparable to our data of 59.3% of all BM patients with significant psycho-oncological burden.It has been reported that patients with primary brain tumors experience unmet supportive care needs, especially in the psychological domain (37). Our work indicates that patients with BM also present with a high level of psycho-oncological distress that requires adequate intervention. Tumor-induced symptoms and impairments as well as tumor-targeted treatments may affect one’s ability to carry out daily routine tasks, resulting in increased functional dependency, significant emotional distress, and anxiety about the future (38). Distress in cancer is a multifactorial unpleasant experience that results in the loss of the patient’s coping strategies (39). This statement indicates that the topic of psycho-oncological support comprises a comprehensive set of complex issues that require multidisciplinary, disease-specific experience. Our purpose, however, was to evaluate correlations between psycho-oncological needs and specific aspects to identify patients most in need of support in a well-defined study population.

There is widespread evidence that physical symptoms of specific types of cancer may contribute to depression (40). Among all preoperative functional symptoms, only aphasia was shown to be significantly associated with higher levels of psycho-oncological distress in our data. In our univariate analysis, the other factors related to higher psycho-oncological burden were synchronous metastasis, tumors in eloquent areas, lower KPS, and female gender. Concerning the role of the KPS, some authors did not find any correlation between KPS and psycho-oncological needs (10, 37), while other studies are in line with our data (41, 42). In our study, age was no relevant factor for psycho-oncological burden, which is consistent with similar reports (10). The relationship between age and psychological burden in cancer patients is controversial in the literature: some studies have shown that younger patients are more likely to experience psychological issues and have a higher frequency of anxiety symptoms than older patients (43, 44). However, other studies have indicated that cancer patients over 85 years of age are more likely to develop depression than younger patients (45, 46).

Excluding possible confounders, synchronous metastasis timing, KPS, and female gender were factors associated with a higher risk of psycho-oncological burden. The fact that patients with synchronous metastasis have a higher psycho-oncological distress seems reasonable, considering that the impact of a diagnosis of brain metastasis in patients who have already known about the primary tumor for at least 3 months may be different from that in patients who receive a diagnosis of BM and a diagnosis of primary tumor at the same time or within a very short interval.

### 4.2 Role of gender in psycho-oncological burden

Several studies have already identified female gender as a significant risk factor for higher psycho-oncological burden in cancer patients and have shown that this subpopulation experience more psychological distress than male patients (47, 48). Rapp et al. also identified female gender as a factor associated with a higher risk of pathological screening in both univariate and multivariate analyses (3). Some authors have indicated that even the gender of the caregivers predicted a higher burden (49, 50) and that the level of QoL in female patients was lower than that of male patients (50). These findings are in line with other studies analyzing QoL in different types of cancer: for example, female patients with chronic lymphocytic leukemia were found to have remarkably lower QoL scores in the areas of emotional and social functioning than male patients (51). Few studies have found no association between gender and the prevalence of depression, anxiety, or psycho-oncological needs (5, 37), while other authors suggest the opposite (52, 53), finding anxiety and depression more common in male patients (54). In a recent review, Zhou et al. (2023) stated that gender differences go beyond the simple masculine-feminine binary (55). According to other authors’ findings, the impact of gender on distress, anxiety, and depression is still inconclusive when other factors, such as the primary tumor type and level of education, are considered (56). Other key factors also play a role in the development of psycho-oncological distress, for example, the presence of pre-existing mental health problems and their severity, healthcare costs, access to welfare support, as well as fewer educational qualifications and lack of social support (6).

An unambiguous, scientific explanation of why female patients tend to have a higher psycho-oncological burden is currently not possible. Considering the experience of our center, we can speculate that women tend to communicate their needs and problems more transparently than male patients, who often prefer not to show any signs of suffering. This possible interpretation is reflected in the considerations by Northouse et al. (2000), who maintained that female patients are more comfortable disclosing their emotional distress and role problems. However, they are responsible for managing more roles inside and outside of the family and hence experience more role disruption and distress when illness occurs (49). This concept is reinforced by the fact that although female patients were more likely to experience depression, male patients were more likely to experience somatization (57).

In our opinion, these findings and considerations underscore two critical needs in the management of patients with brain metastasis. First, a gender-sensitive approach in psycho-oncological support, as already recommended by some authors (49, 58), and second, to provide other psychological support strategies for male patients, considering that their psycho-oncological distress may be underestimated due to possible psychological embarrassment, reluctance to bother the physician, and higher barriers to help-seeking (59–62).

### 4.3 Possible supporting strategies to enhance quality of life

Once the causes of increased psycho-oncological needs have been identified, it would be appropriate to develop a strategy to reduce this burden (63). Notably, the use of psycho-oncological interventions in other oncological diseases can reduce psychological burden and improve QoL compared to patients receiving standard support alone (64). Effective psychotherapy for depression in patients with brain tumor is limited compared with cognitive behavioral therapy and participation in support groups (65). Therefore, an accurate identification of the categories of patients most in need of psycho-oncological support, who are carefully sensitized to targeted behavioral strategies, may lead to a breakthrough in the treatment of patients and improve a patient-centered healthcare service delivery model that helps individuals overcome barriers (66).

As more and more patients live with and beyond the diagnosis of BM, more research is needed to understand the potential impact of the long-term and late effects of cancer treatment on mental health and to prevent psycho-oncological burden. The treatment of co-morbid depression and anxiety in people with cancer requires higher clinical priority (6). A better understanding of the correlates of existential tension in patients with brain tumor is essential (65), and will ultimately improve patient-centered care (67) and address the quality of survival in addition to quantity (38).

As the prevalence of BM is steadily increasing and surgical success significantly affects prognosis by making adjuvant treatment more effective (68), neurosurgeons will be in contact with an increasing number of patients with brain metastases. Therefore, their respective departments should be prepared to recognize and adequately approach the essential psycho-oncological aspect as well.

### 4.4 Limitations

Our study has several limitations. The first limitation is the single-center, cross-sectional setting. Our data were, in fact, collected at a single point in time, so we cannot verify how the patients’ needs evolved over time. This aspect will be analyzed by our group in a subsequent study. Moreover, due to the number of possible interactions, we did not investigate every single possible factor associated with mental health in general and in gender in particular. This problem is confirmed by other studies in the literature (69). In line with other authors (70), the level of psycho-oncological distress in each phase of care and the specific proposal for support and its effectiveness need to be clarified in further studies.

## 5 Conclusion

Our results show that the majority of BM patients experience a high level of psycho-oncological distress. In the multifactorial analysis, female gender, presurgical KPS, and synchronous BM presentation resulted as independent factors associated with a higher psycho-oncological burden and a major need for psycho-oncological intervention. The task of the treating physician should be to identify individuals with higher psycho-oncological needs in advance and to actively address their needs with a personalized, patient-centered approach to minimize the patients’ psycho-oncological burden and to improve QoL.

## Data Availability

The raw data supporting the conclusions of this article will be made available on reasonable request via the corresponding authors.
