# Supplementary material for: Psycho-oncological burden in patients with brain metastases undergoing neurological surgery
Source: Front Oncol. 2024 Nov 28;14:1463467. doi: 10.3389/fonc.2024.1463467 (PMC11634795; doi:10.3389/fonc.2024.1463467)
Supplement: Supplementary file 1 [file Table1.docx]

**Supplementary Material**

**A.**


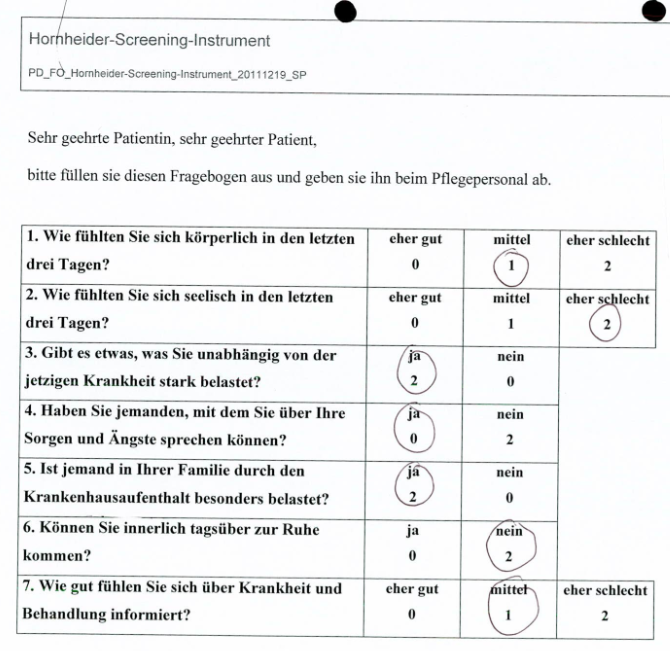


Exemple of a fulfilled Hornheider Screening Instrument (HSI) questionnaire in German language. Considering the sum of the seven items, the score is 10, thus indicating a clear high level of psycho-oncological distress.

The seven items investigate global health, global mental condition, burden, person of trust, burdened family member, temporary internal disturbance, and information about the disease and treatment. The single items are aggregated into a summary score ranging from 0 to 14. The cut-off is set at 5 score points, with scores ≥ 5 points indicating need for psycho-oncological support.

The seven questions can be translated as follows:

“How did you feel physically in the last three days?”

“How did you feel mentally in the last three days?”

“Is there anything that is weighing heavily on you, regardless of the current illness?”

“Do you have someone you can talk to about your worries and fears?”

“Is anyone in your family particularly burdened by your stay in hospital?”

“Are you able to calm down during the day?”

“How well informed do you feel about your illness and treatment?”

**B.**


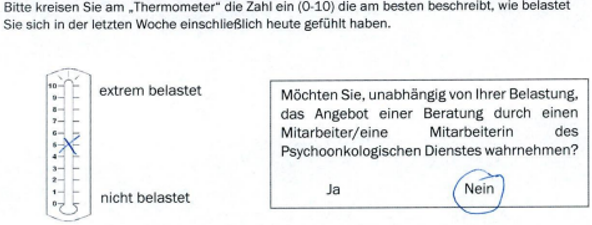


Example of a fulfilled distress thermometer (DT) questionnaire in German language. The thermometer shows a value of 5, thus above 4, established as the threshold for detecting psycho-oncological burden.

Interestingly, it can be seen that in the box on the right, the patient indicates no need to be in contact with the psycho-oncological service of the facility despite high psycho-oncological distress.

The text reads as follows:

(left) Please circle the number on the “thermometer” (0 = not distressed - 10 = extremely distressed) that best describes how burdened you have felt in the last week, including today.

(right) Would you like to accept the offer of counseling by an employee of the psycho-oncology service, regardless of your stress level? “yes”, “no”.
